# Risk Factors Associated With Neurological and Extra-Neurological Complications and Mortality in Patients With Stroke

**DOI:** 10.7759/cureus.40706

**Published:** 2023-06-20

**Authors:** Héctor A Rodríguez-Rubio, Rodrigo López-Rodríguez, Jonathan Ramos-Escalante, Alfredo Bonilla-Suastegui, Juan Carlos Balcázar-Padrón, Luis A Rodríguez-Hernández, Carlos F Nicolas-Cruz, Fernando Piñón-Jiménez, Miguel Angel Siller Uvalle, Aleida Arritola-Uriarte, Alejandro Leal-Galvan, Bill R Ferrufino-Mejia

**Affiliations:** 1 Neurological Surgery, Instituto Nacional de Neurología y Neurocirugía "Manuel Velasco Suárez", Mexico City, MEX; 2 Neurology, Hospital General de Tampico "Dr. Carlos Canseco", Tampico, MEX

**Keywords:** stroke mortality, ischemic stroke, hemorragic stroke, risk factors, cerebrovascular stroke

## Abstract

Introduction

Understanding when and how systemic complications can occur after an acute stroke is crucial. It is important to identify patients who are at higher risk for these complications. Early and effective treatment based on this knowledge can significantly improve patient outcomes. The objective of this study was to identify the risk factors associated with neurological and extra-neurological complications and mortality in stroke patients treated at a secondary care hospital.

Methods

Of a total of 170 patients diagnosed with hemorrhagic/ischemic stroke and transient cerebral ischemia at a secondary care hospital in Mexico, the records of 125 were reviewed and of these, 86 were included in the study. The study group comprised 86 adult patients (> 18 years of age) diagnosed with ischemic or hemorrhagic stroke or transient cerebral ischemia. Their demographics, clinical characteristics, in-hospital complications, and mortality were retrospectively analyzed.

Results

Of the 86 patients examined, 34.9% experienced complications, regardless of the type of stroke. The most significant factor associated with mortality and complications during hospitalization in patients with stroke was previous diseases. Other factors that were linked to higher mortality were pre-existing medical conditions. The most common neurological complication among patients with stroke during hospitalization was intracranial hypertension (3.5%). As for extra-neurological complications, pressure ulcers and nosocomial pneumonia had an occurrence rate of 4.7%.

Conclusions

The main neurological complication during hospitalization of patients with stroke was intracranial hypertension, while the extra neurological complications were pressure ulcers and nosocomial pneumonia.

## Introduction

Cerebrovascular disease is a leading global cause of death and can result in significant short and long-term disability. Almost one-third (30.6%) of patients experience immediate complications while staying in the hospital. It's important to note that there is also a possibility of late events occurring after discharge, interfering with the patient's quality of life [1]. If a stroke is detected early, many complications that may occur during hospitalization can be prevented or treated [2]. 

Complications such as endocranial hypertension, fever, pain, stroke progression, and infections are common. However, there is also a risk of experiencing myocardial infarction, pulmonary embolism, and cardiac arrest [3,4]. Therefore, identifying risk factors associated with complications in stroke is essential for their correct management [5]. The most frequent risk factor related to cerebrovascular events is high blood pressure, a sedentary lifestyle, and obesity [6]. During the last decades, the stroke population has also changed, with a higher proportion of mild strokes probably due to better primary prevention [7]. Mortality is related to infectious pneumopathies, cerebral edema, and pulmonary thromboembolism [8]. However, there are extra neurological complications of which hyponatremia and hypoglycemia have been found more frequently in multiple studies [9]. The risk of complications is strongly related to the severity of the stroke. Hence, knowledge of the most frequent complications justifies the protocolization of care and close patient monitoring to detect, treat, and prevent these events [10-12].

## Materials and methods

Between January and December 2021, 170 patients were diagnosed with hemorrhagic/ischemic stroke and transient cerebral ischemia at a secondary care hospital, Tampico General Hospital "Dr. Carlos Canseco", in Tampico, Mexico. Inclusion criteria were adult patients (> 18 years of age) of both genders diagnosed with ischemic or hemorrhagic stroke or transient cerebral ischemia. Exclusion criteria were a history of previous strokes, individuals totally dependent on others for daily activities before the stroke event, patients with pneumonia, neuromuscular alterations, and pressure ulcers before the stroke, and patients with psychiatric pathologies before the stroke event.

The study was based on clinical records and neuroimaging studies of patients treated at the Department of Neurology of Tampico General Hospital "Dr. Carlos Canseco" during the study period. Out of the 170 total patients, the files of only 125 were examined as 45 were not found. Of the 125 files, 39 were excluded for various reasons; four didn't meet the inclusion criteria, three had a different diagnosis confirmed after admission, 14 had missing data (such as scales or imaging reports), five took voluntary discharge, and 13 had incomplete records. The study group ended up consisting of 86 records.

We analyzed the patients' epidemiological characteristics and clinical history, which included their comorbidities, baseline measurements, stroke presentation, risk factors, assessment measures of neurological function using the National Institutes of Health Stroke Scale (NIHSS), complications, and hospital mortality. During the hospital stay, the presence or absence of complications was analyzed based on the type of cerebrovascular disease. The complications were divided into two groups: neurological and extra-neurological. We also evaluated the patient's history and risk factors for stroke, including body mass index (WHO classification), high blood pressure, diabetes, smoking, exposure to biomass, alcohol consumption, history of drug addiction, and past severe acute respiratory syndrome coronavirus 2 (SARS-CoV-2) infection.

The IBM SPSS Statistics for Windows, Version 25.0 (Released 2017; IBM Corp., Armonk, New York, United States) was used. The results were analyzed by a binary logistic regression analysis model. Odds ratio (OR) and 95% confidence intervals (CIs) of each variable were estimated. Interaction terms between variables were examined using the Pearson Chi-square, Fisher’s exact test, and t-test. A P-value < 0.05 was considered to be significant.

## Results

The mean age of the 86 study subjects was 63 years (range 27-89 years), and the distribution by gender was 47.7% (n=41) females and 52.3% (n=45) males. A distribution by type of cerebral vascular event was made: 61.6% (n=53) were ischemic, 22.1% (n=19) were hemorrhagic, and 16.3% (n=14) were transient ischemic. Of these, only 53.5% of the patients had an NIHSS measurement. The most frequent pathologic antecedents were: high blood pressure 82.6% (n=71), diabetes 57% (n=49), secondary disease 41.9% (n=36), overweight 40.7% (n=35), class 1 obesity 37.2% (n=32), smoking 32.6% (n=28), alcohol consumption 31.4% (n=27), exposure to biomass 20.9% (n=18), class 2 obesity 8.1% (n=7), drug addiction 2.4% (n=2), SARS-CoV-2 infection 2.4% (n=2), and class 3 obesity at 1.2% (n=1).

Table 1 shows in detail the risk factors observed globally. Arterial hypertension and diabetes mellitus predominate as antecedents in ischemic and hemorrhagic presentations. In transient ischemic events, arterial hypertension and overweight were the most common comorbidity. Regarding concomitant diseases, hypertensive emergency was reported in 15% (n=17.4) of patients, uncontrolled diabetes in 7% (n=8.1), and atrial fibrillation, ischemic heart disease, and dyslipidemia in 6% (n=7).

**Table 1:** Distribution of risk factors in patients with stroke SARS-Cov-2: severe acute respiratory syndrome coronavirus 2

Comorbidities	Total % (n=86)	Ischemic % (n=53)	Hemorrhagic % (n=19)	Transient ischemic % (n=14)
Overweight	40.7% (35)	39.6% (21)	31.6% (6)	57.1% (8)
Class 1 obesity	37.2% (32)	23.3% (20)	9.3% (10)	4.7 % (4)
Class 2 obesity	8.1% (7)	4.7% (4)	3.5% (3)	0% (0)
Class 3 obesity	1.2% (1)	1.2% (1)	0% (0)	0% (0)
Secondary disease	41.9% (36)	25.6% (21)	12.8% (11)	3.5% (3)
High blood pressure	82.6% (71)	50% (41)	20.9% (18)	11.6% (10)
Diabetes mellitus	57% (49)	38.4% (33)	10.5% (9)	8.1% (7)
Smoking	32.6% (28)	18.6% (15)	8.1% (7)	5.8% (5)
Alcohol consumption	31.4% (27)	16.3% (13)	9.3% (8)	5.8% (5)
Biomass exposure	20.9% (18)	14% (11)	2.3% (2)	4.7% (4)
Drug addiction	2.4% (2)	1.2% (1)	1.2% (1)	0% (0)
SARS-Cov-2 infection	2.3% (2)	2% (1)	100% (1)	0% (0)

Predictors of mortality

Hospital mortality was 10.5%. Table 2 shows the variables that were independently associated with higher mortality. Those with significant differences (p<0.05) were: secondary disease (OR =14; 95%CI = 1.667-27.817), presence of comorbidities (OR =9.9; 95%CI = 1.912-15.042), BMI (OR =3.1; 95%CI = 1.564-6.953), high blood pressure (OR =1. 7; 95%CI = 1.205-5.385) and diabetes mellitus (OR =1.5; 95%CI = 1.368-6.789). Variables with a non-significant difference (p>0.05) were: age (OR =0.42; 95%CI = 0.233-8.923), smoking (OR=1.767; 95%CI 0.435-7.167), and biomass exposure (OR =3.1; 95%CI = 0.463-9.228).

**Table 2:** Risk factors and factors associated with mortality in patients with stroke

Factor	Total % (n=86)	Deaths % (n=9)	Survivors % (n=77)	OR	95%CI	P-value
Sex				0.87	0.216-3.468	1.00
Female	41 (47.7%)	4 (44.4%)	37 (48.1%)			
Male	45 (52.3%)	5 (55.6%)	40 (51.9%)			
Secondary disease	36 (41.9%)	8 (22.2%)	28 (77.8%)	14	1.667-27.817	0.00
Comorbidities	29 (30.5%)	7 (24.1%)	22 (75.9%)	9.94	1.912-15.042	0.00
Overweight	29.75±4.04	33.56±2.74	29.30±3.94	3.16	1.564-6.953	0.00
High blood pressure	71 (82.6%)	8 (11.3%)	63 (88.7%)	1.78	1.205-5.385	0.00
Age	63.43±13.2	61.6 ± 12.3	63.6 ± 13.6	0.42	0.233-8.923	0.72
Diabetes	49 (57%)	6 (12.2%)	43 (87.8%)	1.58	1.368-6.789	0.03
Smoking	28 (32.6%)	4 (14.3%)	24 (85.7%)	1.77	0.435-7.167	0.18
Biomass exposure	18 (20.9%)	3 (16.7%)	15 (83.3%)	2.07	0.463-9.228	0.38
Alcohol consumption	27 (31.4%)	5 (18.5%)	22 (81.5%)	3.13	0.767-12.732	0.14

Predictors of complications

A total of 34.9% (n=30) of patients had complications up to the time of discharge, of which 40% (n=12) were neurological, and 60% (n=18) were extra-neurological. The variable independently associated with a more significant number of neurological and extra-neurological complications was secondary disease (OR = 3.9; 95%CI = 1.554-10.102, p <0.05) and class 1 obesity (OR = 4.4; 95%CI = 2.88-5.99). The rest of the variables are summarized in Table 3.

**Table 3:** Risk factors and factors associated with complications in patients with stroke

FACTORS	Total % (n=86)	With complications %	Without complications %	OR	95%CI	P-value
Sex				0.62	0.253-1.525	0.66
Female	41 (47.7%)	12 (14%)	29 (33.7%)			
Male	45 (52.3%)	18 (20.9%)	27 (31.4%)			
Secondary disease	36 (41.9%)	19 (22.1%)	17 (19.8%)	3.96	1.554-10.102	0.01
Class 1 obesity	29.75±4.04	31.85±4.08	28.24±3.07	4.44	2.883-5.994	0.04
High blood pressure	71 (82.6%)	24 (27.9%)	47 (54.7%)	0.77	0.244-2.405	0.20
Age	63.43±13.2	63±13.2	65±13.6	0.55	0.32-6.07	0.88
Diabetes	49 (57%)	29 (33.7%)	20 (23.3%)	1.86	0.740-4.684	0.18
Smoking	28 (32.6%)	11 (12.8%)	17 (19.8%)	1.33	0.521-3.386	0.35
Biomass exposure	18 (20.9%)	6 (7%)	12 (14%)	0.92	0.305-2.751	0.06
Alcohol consumption	27 (31.4%)	11 (12.8%)	16 (18.6%)	1.45	0.564-3.713	0.59

**Figure 1:**
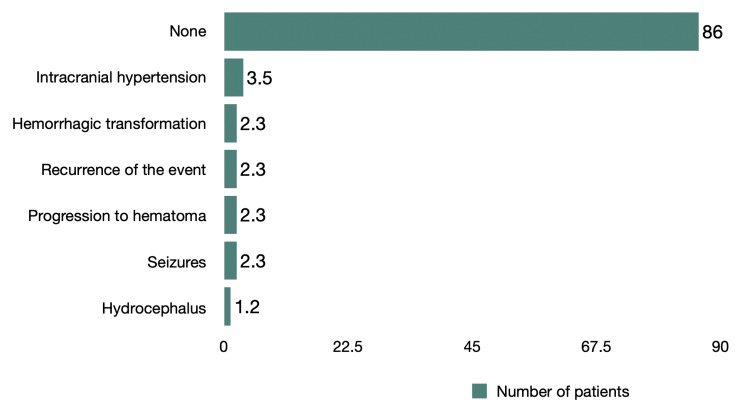
Neurological complications in acute stroke

The main neurological complications were intracranial hypertension at 3.5% (n=3) as well as hemorrhagic transformation, recurrence of the event, progression to a hematoma, and seizures with 2.3% (n=2) each. Only 1.2% (n=1) presented hydrocephalus secondary to a hemorrhagic event (Figure 1).

**Figure 2:**
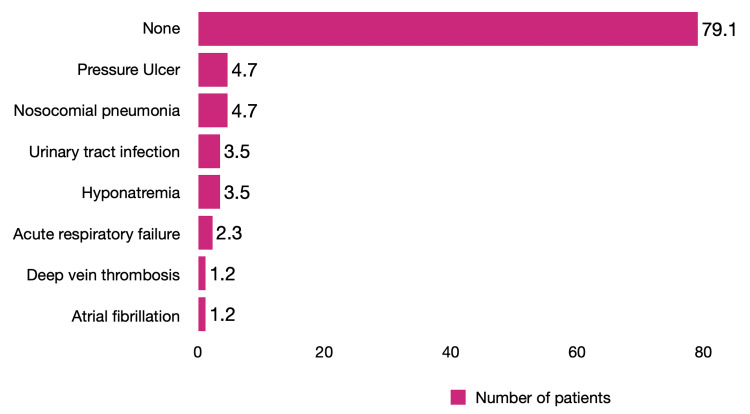
Extra-neurological complications in acute stroke

Moreover, the main extra-neurological complications were: pressure ulcer and pneumonia 4.7% (n=4), urinary tract infection, and hyponatremia 3.5%, (n=3) each. Acute respiratory failure occurred in only 2.3% (n=2), atrial fibrillation, and deep vein thrombosis in 1.2% (n=1) (Figure 2).

## Discussion

The study integrated the analysis of 86 files of adult patients (> 18 years of age) diagnosed with ischemic or hemorrhagic stroke or transient cerebral ischemia. The study was based on clinical records and neuroimaging studies of patients treated at the Department of Neurology from January to December, 2021. The highest prevalence corresponds to the male sex (52.3%), which coincides with other studies; Ekker et al. stated that the prevalence distribution by gender is higher in males (52.7%) [13]. According to other studies, the prevalence distribution tends to be greater in females as the age under investigation increases [14,15]. Our research showed no statistically significant differences concerning the different ages of presentation. Seizures, progression to a hematoma, intracranial hypertension, and transformation to hemorrhagic were the most common neurological complications. Hematoma progression can be partially predicted by assessing imaging parameters (e.g., spot sign) and adequately treating arterial hypertension in the hyperacute phase. Early vascular event recurrence presented a rate similar to that described in the literature analyzed [16]. The principal extra-neurological complications were pressure ulcers, pneumonia, urinary tract infection, and hyponatremia. These data agree with the literature [17-19]. Considering that more than half of the patients (57%) have diabetes mellitus as a comorbidity, it has been shown that early detection and correction of hyperglycemia during the acute phase of stroke improves the prognosis of patients [16]. 

Identifying risk factors associated with stroke complications is essential for their correct management and impacts patient prognosis [16]. The risk factor most frequently associated with stroke events has been arterial hypertension, a sedentary lifestyle, and obesity. According to the findings in the clinical records, we used the phenotypic classification suggested by the Mexican clinical practice guidelines for stroke subtypes; Herath et al. used a division only as hemorrhagic and ischemic stroke [20,21]. 

The most common comorbidity in the ischemic cerebrovascular event was diabetes mellitus with 64% of the cases; in the hemorrhagic event, it was arterial hypertension with 90.5%. The study conducted by Cipolla and her team explored the relationship between hypertensive disorders and stroke. They discovered that these disorders increase shear stress, cause endothelial dysfunction, and stiffen the large arteries that transmit pulsatile flow to the cerebral microcirculation. Additionally, hypertensive disorders contribute to cerebral small vessel disease through hypoperfusion, decreased autoregulatory capacity, and localized increased blood-brain barrier permeability [22]. 

Hospital mortality is a frequent factor in different studies. A study conducted by Labán-Seminario et al. analyzed over 90,000 Ministry of Health records from various health centers. The study found that subarachnoid hemorrhage, intracerebral hemorrhage, and unspecified types of stroke were associated with a higher risk of in-hospital mortality than ischemic stroke. Even after adjusting for age and sex, the risk estimates remained consistent for each type of stroke [23,24].

Public health policies should be improved to reduce the occurrence and death rates related to strokes. This can be done by distributing resources and reorganizing the healthcare system to ensure patients receive appropriate prevention measures without overburdening resources. Additionally, increasing the general population's health education can help people identify early warning signs of strokes and seek prompt medical attention.

## Conclusions

Previous disease in hospitalized patients with stroke is the most associated variable with mortality and complications during hospitalization. Other factors associated with higher mortality were previous comorbidities and being overweight; likewise, obesity grade 1 is a risk factor for complications. The main neurological complication during hospitalization in patients with stroke was intracranial hypertension, and the extra-neurological complication was pressure ulcer and nosocomial pneumonia. Knowledge of the most frequent complications helps the protocolization of care and close monitoring of the patient to detect, treat and prevent them. The ideal setting would be caring for patients in the intensive care unit.
